# Psychological mechanisms of AI integration in ESL teaching: teacher self-efficacy and classroom practice in Ghanaian senior high schools

**DOI:** 10.3389/fpsyg.2026.1851325

**Published:** 2026-06-10

**Authors:** Heikki Opoku Ofori, Emre Debreli

**Affiliations:** Faculty of Education, Cyprus International University, Via Mersin 10, Nicosia, Northern Cyprus, Türkiye

**Keywords:** AI in education, ESL teaching, psychology of language teaching, self-efficacy, Technology Acceptance Model

## Abstract

The integration of artificial intelligence (AI) into language education has gained increasing attention, yet research has predominantly focused on student outcomes, with limited attention to teachers’ roles in mediating these technologies. This study investigates the psychological mechanisms underlying ESL teachers’ integration of AI tools in reading and writing instruction in Ghanaian senior high schools. Grounded in the Technology Acceptance Model and teacher self-efficacy theory, the study adopts a qualitative research design drawing on semi-structured interviews, classroom observations, and stimulated recall interviews. The findings indicate that while teachers generally perceive AI tools as useful and moderately easy to use, these perceptions do not directly translate into pedagogical practice. Instead, teacher self-efficacy emerges as a central psychological mechanism shaping the extent and depth of AI integration in classroom contexts. Teachers with higher confidence demonstrate more structured and pedagogically meaningful use, whereas others rely on limited, surface-level applications. The findings also reveal a persistent gap between positive attitudes and enacted practice, shaped by contextual constraints such as limited infrastructure and time, as well as teachers’ capacity to adapt AI tools within instructional processes. The study contributes to AI in education research by emphasizing the role of teacher psychology as a mediating layer between technological potential and instructional adaptation, offering a more nuanced understanding of how AI-supported practices are enacted in resource-constrained educational contexts.

## 1 Introduction

The rapid advancement of AI has begun to transform educational practices across a wide range of disciplines, including language education. Recent developments in generative AI tools have expanded the possibilities of technology-enhanced learning by enabling more interactive, adaptive, and feedback-driven instructional processes. In the context of English as a second language (ESL) teaching, AI-supported tools such as automated writing evaluation systems and conversational agents have been shown to support language development and provide new opportunities for individualized learning (Zawacki-Richter et al., 2019; Holmes et al., 2019). As a result, AI is increasingly positioned as a significant component of contemporary educational innovation. Despite this growing interest, existing research has largely focused on student outcomes, including improvements in writing quality, reading comprehension, and engagement (Rad et al., 2024). Systematic reviews of AI in education indicate that the majority of studies examine the effectiveness of AI tools on learners, while comparatively fewer studies investigate the role of teachers in mediating these technologies (Zawacki-Richter et al., 2019). This imbalance is problematic because teachers play a central role in determining how technologies are selected, implemented, and adapted within classroom contexts (Moltudal et al., 2022). The successful integration of AI into educational practice is therefore not only a matter of technological capability but also of teacher-related factors.

Recent research highlights the importance of psychological constructs in understanding teachers’ adoption of emerging technologies (Şahin and Şahin, 2022). In particular, teacher self-efficacy has been identified as a critical determinant of technology integration (Williams et al., 2023; Gökalp and Değirmenci, 2025). Teachers with higher levels of self-efficacy are more likely to experiment with innovative tools, persist in the face of challenges, and implement technology in pedagogically meaningful ways (Gökalp and Değirmenci, 2025). Within the context of digital and AI-supported teaching, self-efficacy has been shown to influence both the intention to use technology and the quality of its classroom application (Joo et al., 2018; Alqurni, 2026).

In parallel, the Technology Acceptance Model (TAM) provides a well-established framework for understanding individuals’ adoption of new technologies. According to TAM, perceived usefulness and perceived ease of use are key predictors of technology acceptance and usage (Davis, 1989; Tubaishat, 2018). In educational contexts, numerous studies have confirmed that teachers’ perceptions of usefulness and ease of use significantly influence their willingness to integrate digital tools into their teaching practices (Teo, 2011; Scherer et al., 2019). More recent research suggests that these perceptions are closely linked with teachers’ confidence and prior experience, indicating that psychological and cognitive factors jointly shape technology adoption processes (e.g., Liu, 2025).

While these theoretical perspectives provide valuable insights, research on AI integration in ESL education remains limited in several important ways. First, much of the existing literature is situated in technologically advanced contexts, with relatively little attention paid to under-researched regions such as sub-Saharan Africa. Second, studies that do examine technology use in these contexts often focus on infrastructural challenges and access issues, rather than on the psychological mechanisms that influence teachers’ practices (Tondeur et al., 2017). Third, there is a lack of empirical research that connects teachers’ beliefs, perceptions, and self-efficacy with their actual pedagogical use of AI tools, particularly in language teaching domains such as reading and writing (Huang et al., 2024).

These gaps are especially relevant in the Ghanaian educational context, where efforts to integrate digital technologies into teaching and learning are ongoing but uneven. Structural constraints such as limited access to reliable internet, insufficient training opportunities, and resource disparities may influence teachers’ engagement with AI tools. At the same time, the increasing availability of AI applications creates new possibilities for enhancing ESL instruction. Understanding how teachers perceive, adopt, and implement these tools within such contexts requires close attention to the psychological processes that underpin their decision-making and instructional practices as they are enacted in classroom settings. Addressing this gap is important for both theoretical and practical reasons. From a theoretical perspective, integrating frameworks such as the Technology Acceptance Model and teacher self-efficacy theory allows for a more comprehensive understanding of technology adoption in education by linking cognitive, affective, and behavioral dimensions. From a practical perspective, identifying the factors that support or hinder teachers’ effective use of AI can inform the development of targeted professional development programs and policy initiatives aimed at improving instructional quality. In response to these needs, the present study investigates the psychological mechanisms that influence ESL teachers’ integration of AI tools in reading and writing instruction in Ghanaian senior high schools. Adopting a qualitative approach, the study examines how teachers’ perceptions of AI, their self-efficacy beliefs, and their contextual experiences shape their pedagogical practices and their interpretations of AI’s role in language education. The study is guided by the following research questions:

1. How do ESL teachers perceive the usefulness and ease of use of AI tools in language instruction?
2. How does teacher self-efficacy influence the adoption and use of AI-supported pedagogical practices?
3. How do these psychological factors shape teachers’ classroom practices and perceived instructional outcomes?
4. What contextual challenges influence teachers’ integration of AI tools in Ghanaian senior high schools?

## 2 Literature review

### 2.1 AI integration in ESL teaching

The integration of AI into language education has expanded rapidly in recent years, particularly with the emergence of generative AI tools that support writing, feedback, and interactive learning. In the field of ESL, AI technologies are increasingly used to facilitate key language skills, especially reading and writing. Applications such as automated writing evaluation systems, intelligent tutoring systems, and conversational agents provide learners with immediate feedback, personalized input, and opportunities for extended language practice (Zawacki-Richter et al., 2019; Holmes et al., 2019). These tools are often positioned as enhancing both the efficiency and effectiveness of language instruction by supplementing traditional pedagogical approaches (Kuddus, 2022).

In writing instruction, AI-supported tools such as Grammarly and similar automated feedback systems have been widely adopted to assist learners in improving grammatical accuracy, coherence, and lexical choice (Azennoud, 2024). Research indicates that such tools can support revision processes and promote greater learner autonomy by enabling students to engage with feedback independently (Stevenson and Phakiti, 2019). Similarly, recent studies on generative AI applications, including large language models, suggest that these tools can assist learners during pre-writing and drafting stages by generating ideas, suggesting structures, and modeling language use (e.g., Kasneci et al., 2023). In reading instruction, AI-based platforms have been used to provide adaptive texts, vocabulary support, and comprehension scaffolding, allowing learners to engage with materials that are tailored to their proficiency levels (Holmes et al., 2019).

Despite these promising developments, the existing body of research has predominantly emphasized student-related outcomes. Much of the literature examines how AI tools influence learners’ performance, engagement, and motivation, often through experimental or quasi-experimental designs (Li, 2023). For instance, studies frequently measure improvements in writing quality, reading comprehension, or vocabulary acquisition following exposure to AI-supported instruction (Zawacki-Richter et al., 2019; Kasneci et al., 2023). While such findings provide valuable insights into the potential benefits of AI, they offer limited understanding of how these tools are actually implemented in classroom settings.

In contrast, the role of teachers in mediating AI integration has received comparatively less attention. This is a significant limitation, as teachers are central to shaping how technologies are selected, adapted, and embedded within pedagogical practices (Abedi, 2024). Research on technology integration more broadly has consistently shown that the success of digital innovations depends not only on the availability of tools but also on teachers’ beliefs, competencies, and instructional decisions (e.g., Tondeur et al., 2017). Without considering these factors, it is difficult to explain variations in how AI is used across different contexts or why similar technologies may produce different outcomes. The limited focus on teachers is particularly evident in studies conducted in under-researched contexts such as sub-Saharan Africa. While there is growing recognition of the importance of digital technologies in improving educational quality in these regions, empirical research often concentrates on infrastructural challenges, including access to devices, internet connectivity, and institutional support (Timotheou et al., 2023). Although these factors are undoubtedly important, they do not fully capture the complexities of technology integration, which also involve cognitive and psychological dimensions related to teachers’ perceptions and practices. Furthermore, within ESL education specifically, there is a lack of research that examines how teachers integrate AI tools into the teaching of reading and writing as distinct but interrelated skills. Existing studies tend to treat language learning as a general domain or focus on isolated tools rather than on pedagogical processes (Hung et al., 2018). As a result, there is limited understanding of how AI-supported instruction is enacted in classroom practice, particularly in relation to teachers’ decision-making and instructional strategies.

These gaps suggest that current research provides an incomplete picture of AI integration in ESL education. While the potential of AI to support language learning is well documented, less is known about how teachers interpret, adopt, and implement these tools in real educational settings. This limitation is especially pronounced in contexts such as Ghana, where both opportunities and constraints shape the use of emerging technologies. Addressing this gap requires a shift in focus from student outcomes alone to the psychological and pedagogical factors that influence teachers’ engagement with AI in language instruction.

### 2.2 Psychological determinants of technology adoption

Understanding how teachers integrate AI into their instructional practices requires attention to the psychological factors that influence technology adoption. While access to tools and institutional support are important, research consistently shows that teachers’ beliefs, perceptions, and confidence play a decisive role in shaping whether and how technologies are used in classrooms. Among the most widely used frameworks for examining these processes are the Technology Acceptance Model (TAM) and teacher self-efficacy theory. Together, these perspectives provide a complementary explanation of teachers’ cognitive evaluations of technology and their perceived capability to use it effectively in pedagogical contexts.

#### 2.2.1 Technology Acceptance Model

TAM, originally developed by Davis (1989), remains one of the most influential frameworks for explaining individuals’ adoption of new technologies. According to TAM, two key factors determine technology acceptance: perceived usefulness, defined as the extent to which an individual believes that a technology will enhance performance, and perceived ease of use, which refers to the degree to which the technology is perceived as effortless to use (Davis, 1989). These perceptions shape users’ attitudes toward technology and their intention to adopt it.

In educational settings, TAM has been widely applied to understand teachers’ adoption of digital tools. Empirical studies have consistently demonstrated that teachers are more likely to integrate technology into their teaching when they perceive it as both useful for instructional purposes and manageable within their existing practices (Teo, 2011; Scherer et al., 2019). For instance, when teachers believe that AI tools can improve feedback quality, save time, or enhance student engagement, they are more inclined to experiment with and incorporate these tools into their lessons (Nazaretsky et al., 2022). Conversely, if technologies are perceived as complex, unreliable, or misaligned with curricular goals, teachers may resist their use despite their potential benefits.

Recent research on AI in education suggests that TAM remains highly relevant in explaining teachers’ responses to emerging technologies. Studies indicate that perceived usefulness is often linked to expectations about improved learning outcomes, while perceived ease of use is associated with familiarity, training, and prior experience with digital tools (Scherer et al., 2019). However, these perceptions are not formed in isolation. They are influenced by contextual factors such as institutional support, access to resources, and professional development opportunities, as well as by individual differences in confidence and prior experience. While TAM provides a strong foundation for understanding initial adoption decisions, it has been critiqued for its limited attention to deeper psychological and pedagogical factors (Sadeck, 2022). In particular, TAM does not fully account for whether teachers feel capable of using technologies effectively in complex classroom environments. This limitation highlights the need to complement TAM with additional constructs that capture teachers’ sense of competence and agency in technology use.

#### 2.2.2 Teacher self-efficacy

Teacher self-efficacy, derived from Bandura’s (1997) social cognitive theory, refers to teachers’ beliefs in their ability to organize and execute the actions required to achieve desired instructional outcomes. In the context of technology integration, self-efficacy reflects teachers’ confidence in their capacity to use digital tools effectively to support teaching and learning. A substantial body of research has established self-efficacy as a key predictor of technology integration in education. Teachers with higher levels of self-efficacy are more likely to adopt innovative practices, persist in overcoming technical and pedagogical challenges, and implement technologies in ways that enhance student learning (Gomez et al., 2022; Sharma and Saini, 2022). In contrast, teachers with lower self-efficacy may avoid using unfamiliar tools or limit their use to superficial applications (Gomez et al., 2022).

In relation to AI, self-efficacy becomes particularly important due to the perceived complexity and novelty of these technologies. Integrating AI tools into ESL instruction often requires not only technical skills but also the ability to evaluate outputs, guide students’ use of AI critically, and align AI-supported activities with pedagogical objectives. Teachers who feel confident in these areas are more likely to use AI in meaningful and sustained ways. Emerging evidence suggests that self-efficacy is positively associated with both the frequency and sophistication of technology use, including the integration of advanced digital tools (Joo et al., 2018; Mekheimer, 2025). Moreover, self-efficacy interacts closely with technology acceptance. Teachers who perceive AI tools as useful and easy to use are more likely to develop confidence in using them, while higher self-efficacy can reinforce positive perceptions of technology (Bergdahl and Sjöberg, 2025). This reciprocal relationship suggests that adoption is not solely driven by external characteristics of the technology but also by teachers’ internal beliefs about their capabilities. However, despite its importance, self-efficacy has often been examined independently of specific pedagogical practices. There is limited research that directly connects teachers’ self-efficacy beliefs with how they design and implement AI-supported instruction in language classrooms (Qiu, 2025). This gap is particularly evident in ESL contexts, where the integration of AI into reading and writing instruction involves distinct pedagogical considerations.

TAM and teacher self-efficacy provide a robust framework for understanding the psychological determinants of AI adoption in education. TAM explains how teachers evaluate the usefulness and usability of AI tools, while self-efficacy captures their perceived ability to translate these tools into effective instructional practices. However, to fully understand AI integration in ESL classrooms, it is necessary to examine how these psychological factors are reflected in actual pedagogical practices and shaped by contextual conditions.

### 2.3 Linking teacher psychology to pedagogical practice

While existing research has established the importance of technology acceptance and self-efficacy in shaping teachers’ adoption of digital tools, there remains limited understanding of how these psychological factors translate into actual classroom practices. Much of the literature treats technology use as an outcome variable, focusing on whether teachers adopt specific tools rather than examining how their beliefs and perceptions shape the ways in which these tools are pedagogically implemented. This distinction is particularly important in the context of AI, where effective use depends not only on adoption but also on the quality and appropriateness of instructional integration.

In AI-supported ESL instruction, this connection becomes especially significant. Teaching reading and writing with AI tools requires teachers to make decisions about when and how to incorporate these tools into different stages of instruction, such as idea generation, drafting, feedback, and revision (Milad et al., 2025). It also involves guiding students in interpreting AI-generated outputs and developing critical awareness of their limitations. These practices extend beyond basic technology use and require the integration of pedagogical knowledge, technological understanding, and professional judgment. Despite these demands, empirical research examining how teachers’ perceptions of AI and their self-efficacy beliefs shape such instructional decisions remains limited. Existing studies often rely on self-reported measures of technology use without exploring how technologies are enacted in classroom practice (Chiu et al., 2025). As a result, there is insufficient understanding of the relationship between teachers’ psychological dispositions and their enacted pedagogy, particularly in language education contexts where instructional processes are complex and context-dependent. This gap is especially relevant in contexts such as Ghana, where both opportunities and constraints influence the use of emerging technologies. Structural factors, including infrastructure, access to reliable internet, and availability of training, shape how AI tools are used in classrooms. However, these contextual conditions interact with teachers’ psychological factors rather than operating independently. Even when teachers perceive AI as useful, their ability to integrate it effectively depends on their confidence in managing instructional and technical demands. These considerations highlight the need for research that moves beyond examining technology adoption as a standalone outcome and instead investigates how teachers’ psychological factors shape their pedagogical engagement with AI tools. By focusing on this relationship, the present study aims to provide a more nuanced understanding of how AI is enacted in ESL classrooms, particularly in under-researched contexts where both psychological and contextual factors play a critical role.

## 3 Theoretical framework

The integration of AI into educational practice is not solely determined by the availability of technological tools but is strongly influenced by teachers’ psychological dispositions. To explain how ESL teachers adopt and implement AI tools in their instructional practices, this study draws on two complementary theoretical perspectives: the Technology Acceptance Model (TAM) (Davis, 1989) and teacher self-efficacy theory (Bandura, 1997). These frameworks provide a coherent basis for understanding how teachers’ perceptions and beliefs shape their engagement with AI in classroom settings.

TAM proposes that individuals’ adoption of a technology is primarily influenced by their perceptions of its usefulness and ease of use. Perceived usefulness refers to the extent to which a teacher believes that a technology enhances instructional effectiveness, while perceived ease of use reflects the degree to which the technology is seen as manageable and not overly complex (Davis, 1989). In educational contexts, these constructs have consistently been shown to influence teachers’ willingness to adopt and integrate digital technologies into their teaching practices (Scherer et al., 2019). In the case of AI-supported ESL instruction, perceived usefulness may relate to expectations that AI tools can support feedback processes, facilitate language development, and enhance student engagement, whereas perceived ease of use may depend on factors such as prior experience, training opportunities, and the usability of specific tools (Zhao, 2025).

While TAM explains how teachers form initial evaluations of technology, it does not fully account for their ability to translate these perceptions into effective pedagogical practice. For this reason, teacher self-efficacy is incorporated as a complementary construct. Teacher self-efficacy refers to teachers’ beliefs in their capability to organize and execute the actions required to achieve instructional goals (Bandura, 1997). In the context of technology integration, self-efficacy has been identified as a key factor influencing both the adoption and the quality of technology use in classrooms. Teachers with higher levels of self-efficacy are more likely to experiment with innovative tools, persist in overcoming challenges, and implement technologies in ways that support meaningful learning (Gomez et al., 2022).

In the case of AI, self-efficacy becomes particularly important due to the complexity and evolving nature of these tools. Integrating AI into ESL instruction requires not only technical skills but also the ability to evaluate AI-generated outputs, guide students’ use of these tools critically, and align AI-supported activities with pedagogical objectives. Teachers who feel confident in their ability to perform these tasks are more likely to move beyond initial adoption and engage in sustained and effective use of AI in their teaching practices (Qiu, 2025).

Drawing on these perspectives, the present study conceptualizes AI integration in ESL instruction as a psychologically mediated process in which teachers’ perceptions of AI tools influence their self-efficacy beliefs, which in turn shape their pedagogical practices. In this framework, perceived usefulness and perceived ease of use are associated with teachers’ confidence in using AI, while self-efficacy is linked to the extent and manner in which AI tools are incorporated into classroom instruction. These pedagogical practices are further related to teachers’ perceptions of instructional outcomes, particularly in relation to the teaching of reading and writing. This integrated framework enables a more comprehensive understanding of AI adoption in educational contexts by linking cognitive evaluations of technology with beliefs about personal capability and instructional behavior. By focusing on these relationships, the study moves beyond examining whether teachers use AI and instead explores how and why they engage with these tools in their pedagogical practice, in line with the research questions guiding the study.

**Figure 1:**
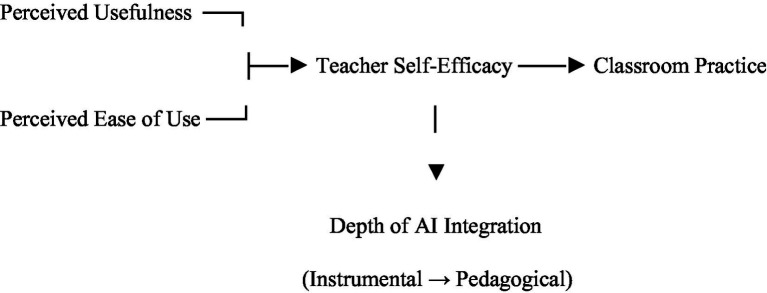
Conceptual framework illustrating the relationship between TAM constructs, teacher self-efficacy, and classroom practice in AI-supported ESL instruction.

In the context of this study, a distinction is made between instrumental use of AI and pedagogical integration. Instrumental use refers to situations in which AI tools are employed primarily to support teacher-centered tasks, such as generating examples or providing quick feedback, without being meaningfully incorporated into student learning processes. In contrast, pedagogical integration involves embedding AI within instructional design in ways that actively engage students in learning activities, such as evaluating, interacting with, and critically reflecting on AI-generated content. This distinction provides an analytical lens for examining variations in how AI is enacted in classroom practice. The relationships among perceived usefulness, perceived ease of use, teacher self-efficacy, and classroom practice are summarized in Figure 1.

## 4 Methodology

### 4.1 Research design

This study adopts a qualitative multiple-case study design aimed at developing an in-depth understanding of the psychological mechanisms underlying teachers’ integration of AI tools in ESL instruction. The multiple-case study approach was considered appropriate as each participating teacher represents a distinct case through which perceptions, practices, and contextual conditions can be examined both individually and comparatively. This design was selected because the study seeks to explore how teachers perceive, interpret, and enact AI-supported practices in real classroom contexts, with particular attention to the role of self-efficacy, perceived usefulness, and perceived ease of use.

A qualitative approach is appropriate for capturing the complexity of teachers’ decision-making processes and contextual constraints, particularly in relation to AI integration in ESL instruction. It allows for detailed examination of how psychological factors are reflected in pedagogical practices and how these practices are shaped by classroom realities. By focusing on teachers’ lived experiences and enacted practices, the design provides both analytical depth and contextual explanation, ensuring alignment with the study’s focus on psychological mechanisms and classroom practice. Furthermore, the use of multiple data sources, including interviews, classroom observations, and stimulated recall interviews, enables triangulation across cases, thereby strengthening the credibility and depth of the analysis. Given the exploratory nature of AI integration in the Ghanaian context and the relatively small sample size, the design does not aim to establish causal relationships but rather to generate contextually grounded insights into teachers’ experiences and practices.

### 4.2 Participants and sampling

The participants in this study consisted of 22 ESL teachers working in Ghanaian senior high schools. A non-probability sampling strategy combining convenience and purposive sampling was employed. This approach was appropriate given the study’s focus on teachers who have at least some familiarity with digital or AI-supported instructional tools, as well as the practical constraints associated with access and participation. In addition to interview participation, a subset of teachers was selected for classroom observations based on their availability, willingness to be observed, and engagement with AI-supported instructional practices.

The sample included both male and female teachers, ensuring basic gender representation. Specifically, 12 participants were male and 10 were female, reflecting a relatively balanced distribution. In terms of teaching experience, participants varied considerably, ranging from 2 to over 15 years of experience. This variation was considered important for capturing diverse perspectives, as prior research suggests that teaching experience may influence both technology adoption and instructional practices (Kearney et al., 2018).

Participants were drawn from different schools within Ghana, providing variation in institutional contexts, including differences in access to technological resources and support. While the sample size is limited, it is adequate for a qualitative study that prioritizes depth of understanding over generalizability. The study does not aim to produce statistically generalizable findings but rather to develop rich, contextually grounded insights that can inform future research and practice in similar contexts.

### 4.3 Data collection methods

#### 4.3.1 Semi-structured interviews

Semi-structured interviews were conducted to explore teachers’ experiences, beliefs, and practices in depth, particularly in relation to their use of AI tools in teaching reading and writing. The interview protocol was designed to align with the study’s theoretical framework and research questions. The use of semi-structured interviews allowed for consistency across participants while also providing flexibility to probe emerging issues. Questions focused on teachers’ perceptions of AI, their confidence in using such tools, the ways in which they integrate AI into their instruction, and the challenges they encounter (see Appendix A).

#### 4.3.2 Classroom observations

Classroom observations were conducted to examine how AI-supported practices are enacted in real instructional settings. A total of 15 teachers participated in the observation phase. Each teacher was observed during one or two instructional sessions, resulting in 21 observed lessons overall. Individual observation sessions lasted approximately 40 to 50 min, corresponding to a full class period, with a total observation corpus of approximately 16 h.

Observations focused on how teachers incorporate AI tools into reading and writing instruction, the nature of teacher and student interactions, and the extent to which AI is integrated into pedagogical processes. Particular attention was given to lesson structure, moments of AI use, teacher guidance, and student engagement with AI-generated outputs. This method enabled the study to move beyond self-reported data and capture actual classroom practices, providing insight into the alignment or mismatch between teachers’ stated beliefs and their instructional behavior (see Appendix B for the observation protocol).

#### 4.3.3 Stimulated recall interviews

Stimulated recall interviews were conducted following classroom observations to gain deeper insight into teachers’ decision-making processes during instruction. These interviews were conducted with all teachers who participated in the observation phase and were based directly on their observed lessons. In these sessions, teachers were invited to reflect on specific moments from their observed lessons and explain their choices, interpretations, and reasoning. This approach allowed for access to teachers’ cognitive and psychological processes as they relate to classroom practice, particularly in relation to confidence, perceived usefulness, and ease of use. It also supported a more nuanced understanding of how teachers interpret and evaluate their use of AI tools in real time.

### 4.4 Data collection procedure

Data collection was conducted in multiple stages. First, semi-structured interviews were carried out to gain an initial understanding of teachers’ perceptions and experiences. Due to participants’ teaching schedules and time constraints, interviews were conducted online at times convenient for the teachers. These interviews lasted approximately 20 to 30 min and were recorded with participants’ consent. Following the interviews, classroom observations were conducted to document teachers’ instructional practices. Out of the 22 participants, 15 teachers were observed in their natural classroom settings. Each of these teachers was observed during one or two lessons, with each session lasting approximately 40 to 50 min. In total, 21 classroom sessions were observed, amounting to approximately 16 h of observation. Observations focused on lessons involving reading and writing activities where AI tools were used or considered. Field notes were taken to capture key aspects of classroom interaction and instructional design. The classroom observations were conducted in person by one of the researchers, who was physically present in the participating schools during the data collection period. No external people were involved in the observation process. To minimize disruption to regular teaching, observations were non-participatory and relied primarily on detailed field notes rather than video recordings. Consequently, stimulated recall interviews were based on these observation notes and teachers’ reflections on specific instructional moments, rather than on video playback. After each observation, stimulated recall interviews were conducted with the observed teachers. These follow-up interviews were conducted online and focused on specific instructional moments identified during the observed lessons. These sessions were used to clarify teachers’ intentions, decisions, and interpretations. All interviews were transcribed for analysis. This multi-stage procedure allowed for triangulation across different data sources, enhancing the depth and credibility of the findings.

Prior to data collection, all ethical procedures were followed, and all participants were informed about the purpose of the study and provided informed consent before participating. Participation was voluntary, and participants were assured of the confidentiality and anonymity of their responses. Data were securely stored and used solely for research purposes.

### 4.5 Data analysis

Qualitative data from interviews, observations, and stimulated recall sessions were analyzed using thematic analysis following the approach outlined by Braun and Clarke (2006). The analysis involved several stages, including familiarization with the data, initial coding, identification of themes, and refinement of thematic categories. The analysis was conducted collaboratively by the two researchers, with the involvement of a third experienced qualitative researcher during the coding and validation stages to enhance analytical rigor and reduce individual researcher bias.

During the familiarization stage, transcripts and observation notes were read repeatedly to identify recurring patterns related to teachers’ perceptions, confidence, and instructional practices. Initial coding was conducted inductively, with segments of data labeled according to their meaning and relevance to the research questions. Coding was first carried out independently by the researchers, after which coding decisions were compared and discussed. Discrepancies were resolved through iterative discussion until consensus was reached, allowing for refinement of code definitions and ensuring consistency in interpretation. For example, statements such as “*I am not sure how to use it during the lesson*” and observed hesitation when introducing AI tools were coded as low instructional self-efficacy, while descriptions of structured AI-supported activities were coded as integrated pedagogical use. Similarly, references to technical difficulty or uncertainty were coded as perceived complexity, and instances where teachers limited AI use to brief demonstrations were coded as instrumental use.

Codes were then grouped into broader categories reflecting relationships within the theoretical framework. This process involved systematically linking initial codes to higher-order categories and subsequently to overarching themes, thereby making explicit the progression from descriptive labeling to conceptual interpretation. For instance, codes related to confidence, hesitation, and risk avoidance were clustered under self-efficacy, while distinctions between surface-level and embedded use informed the category of depth of integration. Through iterative comparison across data sources, these categories were refined into themes such as the perception–practice gap and the role of self-efficacy as a mediating mechanism. To enhance transparency, a summary of the relationships between codes, categories, and themes is presented in Table 1.

**Table 1:** Relationship between codes, categories, and themes.

Initial codes	Categories	Themes
Hesitation, uncertainty, lack of confidence	Self-efficacy (low)	Perception–practice gap
Structured use, planning, guided student interaction	Self-efficacy (high)	Pedagogical integration
Technical difficulty, unfamiliarity	Perceived complexity	Ease of use as a precondition
Brief demonstrations, teacher-only use	Instrumental use	Surface-level integration
Student engagement, evaluation of AI output	Depth of integration	Pedagogical integration
Mismatch between beliefs and practice	Perception vs. enactment	Perception–practice gap

Data from different sources were analyzed both separately and in relation to one another to identify converging and diverging patterns. For example, reported confidence in interviews was compared with observed classroom behavior and further examined through stimulated recall to understand discrepancies between intention and enactment. This process enabled the study to examine how teachers’ reported beliefs relate to their observed practices and how their reflections explain these practices. Triangulation across these data sources was used as a key strategy to enhance the credibility of the findings and to minimize the influence of researcher subjectivity. In addition, themes were reviewed against the full dataset to ensure they accurately represented both dominant and less frequent patterns, including instances that did not fully align with the main trends. Furthermore, the development of themes was based on patterns identified across the full dataset rather than on individual cases alone, ensuring that the analysis reflects both widely shared perspectives and meaningful variations among participants.

## 5 Findings

The findings presented in this section are derived from the full dataset, including all 22 participants, through the integration of interview, observation, and stimulated recall data. While not all participants are quoted directly in each subsection, the themes reflect patterns that were consistently identified across the sample. The selection of illustrative quotes was guided by their clarity, representativeness, and ability to capture key variations in teachers’ experiences and practices. In addition to dominant patterns, attention was also given to less frequent or contrasting cases during analysis to ensure a more comprehensive and balanced interpretation of the data.

### 5.1 The perception–practice disjuncture: usefulness without enactment

Across data sources, a consistent tension emerged between teachers’ recognition of AI’s instructional value and their limited pedagogical enactment. While participants articulated strong beliefs in the usefulness of AI, this did not translate into sustained or structured classroom integration. This pattern reflects a limitation of perceived usefulness as conceptualized in TAM, suggesting that cognitive evaluation alone does not ensure pedagogical uptake. Teachers frequently framed usefulness in abstract or outcome-oriented terms, yet positioned their own practice as constrained or partial:

“For me, the main issue is not whether AI is helpful, because we all know it is helpful. It can really support writing, especially with feedback. But when I am teaching, I hesitate because I am not always sure how to use it effectively in that moment” (T7).

This disjuncture was visible in classroom observations. In several cases, AI tools were introduced briefly during lessons but remained peripheral to instructional design. For example, one teacher used an AI tool to generate a model paragraph but did not integrate it into a broader writing activity or guide students in evaluating the output. Stimulated recall revealed that such decisions were not due to lack of perceived value but to uncertainty about pedagogical application:

“I showed them the example because it is useful, but I did not go deeper. At that moment, I was not confident about how to turn it into an activity, so I moved on” (T7, recall).

These findings indicate that perceived usefulness operates primarily at the level of recognition rather than enactment. Within the theoretical framework, this suggests that TAM constructs alone are insufficient and need to be considered alongside additional psychological mechanisms to influence practice.

### 5.2 Ease of use as a precondition for self-efficacy

Perceived ease of use emerged not as a direct driver of practice but as a condition shaping teachers’ sense of capability. Across interviews and recall data, usability was closely tied to confidence, supporting the view that ease of use indirectly influences pedagogical behavior through self-efficacy.

Teachers consistently described how initial experiences with AI tools shaped their confidence:

“When I first tried it, I was confused and that made me lose confidence. I felt like I could not manage it properly in front of students. But after practicing, I realized it is actually simple, and now I feel more comfortable using it” (T18).

“At first, I found it difficult to navigate the tool, and that made me hesitant to use it during lessons. But as I practiced more, I started to feel more confident and could use it more smoothly with students” (T4).

This relationship was also evident in observed practice. Teachers who demonstrated smoother interaction with AI tools were more likely to incorporate them into lesson sequences, while those who hesitated technically tended to limit use to brief or isolated moments. In stimulated recall, one teacher explicitly linked usability to instructional risk:

“If I feel unsure about how the tool will respond, I avoid using it during the lesson. I cannot take that risk in front of students, because it may disrupt the flow of teaching” (T3, recall).

These findings extend TAM by showing that ease of use functions as a practical enabler of self-efficacy rather than as an independent predictor of use. The data suggest a mediated relationship in which usability shapes confidence, and confidence in turn shapes practice.

### 5.3 Self-efficacy as the mechanism translating perception into practice

Self-efficacy emerged as the central mechanism linking teachers’ perceptions of AI to their pedagogical practices. Teachers with higher confidence demonstrated more deliberate and structured integration, while those with lower confidence relied on fragmented or instrumental use.

In interviews, high-efficacy teachers described AI as embedded within instructional sequences rather than as an auxiliary tool:

“I do not just use AI randomly. When teaching writing, I guide students to generate ideas first, then we evaluate those ideas together. After that, they use AI feedback to revise their drafts, so it becomes part of the lesson process” (T11).

This was corroborated by classroom observations, where such teachers structured activities around AI-supported stages of writing. For example, in one observed lesson, a teacher guided students through a staged writing activity in which learners first generated ideas independently, then compared them with AI-generated suggestions, and finally revised their drafts based on both sources. The teacher actively facilitated discussion around the strengths and limitations of AI outputs, prompting students to justify their choices. Students were engaged not only in using AI outputs but also in discussing and critiquing them. In stimulated recall, these teachers articulated clear pedagogical intentions:

“I wanted them to see that AI is not always correct. That is why I asked them to compare their ideas with the AI output and discuss differences. It helps them think more critically” (T11, recall).

In contrast, teachers with lower self-efficacy described and demonstrated more limited engagement:

“I use it mostly for checking grammar or giving quick examples. I have not really used it as part of a full lesson because I am not fully confident yet. I feel like I still need more experience before I can do that” (T2).

Observed lessons reflected this pattern, with AI used primarily for teacher-centered functions rather than as part of student-centered activities. These findings indicate that self-efficacy plays a central role in shaping how teachers approach the use of AI in classroom practice, particularly in terms of the structure and intentionality of integration.

### 5.4 Self-efficacy as dynamic and situated

Rather than functioning as a stable trait, self-efficacy emerged as a dynamic construct shaped by ongoing interaction with AI tools and classroom contexts. Teachers described confidence as developing through experience, but also as contingent on situational factors.

“At the beginning, I was hesitant because I did not want to make mistakes in front of students. But as I continued using it, I became more confident. Now I can even guide students on how to use it properly and explain its limitations” (T15).

However, observational data indicate that this development is uneven and context-dependent. In some cases, teachers who expressed confidence in interviews reverted to more cautious practices in classrooms with limited connectivity or time pressure. Stimulated recall clarified this shift:

“In that lesson, I had planned to use AI more, but the internet was not stable. So, I decided to reduce its use and focus on the main task instead” (T15, recall).

These findings suggest that self-efficacy operates as both an outcome of experience and a condition for action, but its enactment is closely shaped by contextual constraints. This challenges static interpretations of self-efficacy and highlights its dynamic and context-sensitive nature, emphasizing that teachers’ confidence is continuously negotiated in response to classroom conditions.

### 5.5 From instrumental use to pedagogical integration

While Section 5.3 focused on self-efficacy as a psychological mechanism shaping teachers’ ability to translate perceptions into practice, the present section extends this analysis by examining how this mechanism is reflected in the qualitative differences between instrumental use and pedagogical integration.

A critical distinction emerged between instrumental use of AI and its integration into pedagogical processes. While many teachers used AI to support their own tasks, fewer incorporated it into student learning in a meaningful way.

“Some teachers use it just to save time, like checking or generating content. But I think the real value is when students interact with it and learn how to use it critically, not just rely on it” (T14).

Observational data confirmed this distinction. In one observed case, the teacher used an AI tool solely to generate a model answer, which was presented to students without further discussion or interaction. Students remained passive recipients of the output, and the activity did not extend beyond demonstration. In contrast, other lessons involved students actively engaging with AI responses, comparing alternative outputs, and discussing their appropriateness, reflecting a deeper level of pedagogical integration. In several lessons, AI remained primarily teacher-directed, with minimal student engagement, whereas in others it was incorporated into student-centered learning activities.

Stimulated recall further revealed the reasoning behind these differences:

“If I involve students, I need to be sure I can guide them properly. Otherwise, they may just copy the AI. So sometimes I prefer to use it myself until I feel more confident managing that process” (T9, recall).

This indicates that movement from instrumental use to pedagogical integration is not automatic, but reflects differences in how teachers engage with AI within instructional processes.

### 5.6 Contextual constraints as moderating conditions

The findings demonstrate that psychological factors operate within, and are shaped by, contextual constraints. While teachers consistently recognized the value of AI, infrastructural and institutional limitations influenced how this value could be enacted.

“AI is very helpful for writing lessons, especially for feedback and idea generation. But in reality, we do not always have reliable internet, so sometimes it is difficult to use it during class. That affects how often I can include it in my lessons” (T5).

Time constraints also emerged as a significant factor:

“It is useful, but it requires preparation. If you are already overloaded, it becomes difficult to plan lessons around it. So sometimes you choose simpler methods instead” (T12).

“Sometimes I want to use AI more in my lessons, but the time is not always enough to prepare properly or to guide students through it. So, I end up using it less than I would like” (T6).

Observations confirmed that such constraints often resulted in reduced or simplified use of AI during lessons. However, these constraints did not uniformly determine practice. Instead, their effects were mediated by teachers’ confidence and adaptability, indicating an interaction between psychological readiness and contextual conditions.

The findings suggest that AI integration in ESL classrooms is not solely a function of perception or access, but the result of an interaction between perceived usefulness, ease of use, self-efficacy, and contextual realities. Within this process, self-efficacy emerges as a key mechanism through which perceptions are translated into practice, while contextual factors shape the conditions under which this translation occurs.

## 6 Discussion

### 6.1 Teacher psychology as the foundation of AI integration

The findings reinforce the view that AI integration in ESL instruction is fundamentally a psychologically mediated process, rather than a purely technological or infrastructural one. Consistent with the TAM, teachers’ perceptions of usefulness and ease of use were evident across accounts of AI engagement. However, the qualitative analysis indicates that these perceptions function less as direct drivers of practice and more as preconditions for psychological readiness, particularly in shaping self-efficacy as it is enacted in classroom contexts. This interpretation extends prior research on technology adoption in education. While studies such as Teo (2011) and Scherer et al. (2019) emphasize the predictive role of perceived usefulness and ease of use, the present findings suggest that in the context of AI, these factors appear to operate through teachers’ confidence and their ability to operationalize technology within instructional sequences (Liu, 2025). Teachers frequently articulated strong beliefs about the value of AI, yet observational and recall data revealed hesitation at the point of enactment, indicating that recognition does not ensure pedagogical translation. This aligns with Bandura’s (1997) conceptualization of self-efficacy as a determinant of behavior, but the findings further suggest that self-efficacy must be understood in relation to situated classroom demands. Confidence is not simply a generalized belief but is tied to teachers’ capacity to manage uncertainty, guide student interaction with AI outputs, and integrate tools into ongoing instructional activity. In this sense, the results challenge linear interpretations of TAM and point to a layered process in which cognitive evaluations are filtered through perceived capability before influencing practice, as also suggested in recent work on teacher cognition and technology use (Şahin and Şahin, 2022). Moreover, the findings highlight that psychological readiness develops through interaction with AI tools rather than preceding it. Teachers’ confidence was shaped by prior experiences, including both successful and problematic encounters with AI during instruction. This suggests that teacher cognition is not static but evolves through engagement, supporting the argument that it should be positioned at the center of research on AI integration in pedagogically complex domains such as ESL instruction (Moltudal et al., 2022).

Beyond confirming existing research on technology adoption and self-efficacy, the study contributes by demonstrating how these psychological mechanisms operate within a resource-constrained context, where the translation of positive perceptions into practice is shaped not only by individual beliefs but also by situational limitations. In the Ghanaian context, factors such as inconsistent internet access, limited structured training opportunities, and varying levels of institutional support appear to influence how teachers interpret and enact AI-supported practices. This highlights that the relationship between perception, confidence, and practice is not uniform but contextually conditioned.

### 6.2 The perception–practice gap in AI-supported ESL teaching

A central finding of this study is the persistent gap between teachers’ positive perceptions of AI and their actual pedagogical use of these tools. While teachers consistently recognized the usefulness of AI, qualitative evidence indicates that classroom implementation remained selective, cautious, and often limited to peripheral uses. This reinforces the distinction between acceptance and integration, suggesting that positive evaluation does not necessarily lead to instructional transformation, a pattern also observed in recent studies of AI use in education (Huang et al., 2024). This finding resonates with Ertmer and Ottenbreit-Leftwich (2010), but the present study extends this argument by showing that the gap is not only between belief and practice but also between different levels of practice. Observational and recall data revealed variation between instrumental use, where AI supports teacher tasks, and pedagogical integration, where AI is embedded within student learning processes. Teachers who remained at the instrumental level often framed their decisions in terms of uncertainty and control, indicating that the gap appears to be closely related to perceived capability rather than lack of awareness. The distinction between surface-level and deeper integration is therefore not merely descriptive but reflects underlying psychological differences. Surface use allows teachers to maintain control and minimize risk, whereas deeper integration requires confidence in managing unpredictable outputs, facilitating student interaction, and aligning AI use with pedagogical goals. This aligns with recent research emphasizing that effective AI integration requires not only access but also pedagogical and cognitive readiness (Qiu, 2025; Milad et al., 2025). The findings suggest that many teachers remain at the surface level not because they underestimate AI’s potential, but because they are not fully confident in orchestrating its use within complex classroom environments. This has implications for how AI adoption is conceptualized in the literature. Studies that measure use in binary terms overlook variations in depth and quality, which are critical for understanding instructional impact (Chiu et al., 2025). The present findings indicate that pedagogical transformation requires movement along a continuum of integration, and that this movement is contingent on psychological readiness.

While the dominant pattern reflects a close relationship between self-efficacy and depth of AI integration, some variation was observed. In a small number of cases, teachers who expressed relatively high confidence in interviews demonstrated more limited integration in classroom practice, often due to contextual constraints such as time pressure or technical uncertainty. Conversely, there were instances where teachers with moderate confidence engaged in more active use when conditions were supportive. These variations suggest that the relationship between self-efficacy and practice is not strictly linear but is shaped by situational factors.

### 6.3 Self-efficacy and context as interacting mechanisms

The findings indicate that the translation of AI-related perceptions into pedagogical practice is shaped by the interaction between teacher self-efficacy and contextual constraints. This interaction plays a central role in determining how AI is enacted in classroom settings. However, observational and stimulated recall data show that this process is contingent on contextual conditions such as infrastructure reliability, time availability, and institutional support. This interaction extends prior research on technology integration. While self-efficacy has been consistently identified as a key predictor of technology use (Tschannen-Moran and Hoy, 2001; Teo, 2011), the present findings suggest that its influence is situational rather than uniform, echoing research emphasizing the context-dependent nature of technology adoption (Timotheou et al., 2023; Ifinedo and Kankaanranta, 2021). Teachers who expressed high confidence in interviews did not always enact this confidence in observed lessons when contextual constraints intervened. Conversely, some teachers with moderate confidence were able to integrate AI more effectively when conditions were supportive. These patterns indicate that psychological and structural factors do not operate independently. Instead, self-efficacy functions within contextual boundaries, shaping how teachers respond to constraints rather than eliminating them. In this sense, self-efficacy can be understood as a mechanism that appears to influence whether contextual limitations lead to withdrawal, adaptation, or selective engagement with AI tools, consistent with recent findings on adaptive teacher practices (Gomez et al., 2022; Sharma and Saini, 2022). The Ghanaian context provides an important lens for understanding this interaction. Consistent with Tondeur et al. (2017), infrastructural limitations remain a significant factor in technology integration. However, the findings show that similar constraints do not lead to uniform outcomes. Teachers’ responses varied depending on their confidence, experience, and willingness to experiment, indicating that psychological factors play a critical role in shaping how contextual conditions are experienced and managed (Rad et al., 2024).

The findings support a situated perspective on AI integration in which psychological readiness and contextual conditions are intertwined. AI use in ESL classrooms emerges not as a direct outcome of perception or access, but as a process shaped by the interaction between belief, confidence, and the realities of classroom practice. This perspective provides a more comprehensive framework for understanding variability in teachers’ engagement with AI and highlights the need for interventions that address both psychological and contextual dimensions simultaneously. In addition, the study’s methodological rigor is supported by the systematic validation of themes through iterative coding, cross-checking among researchers, and triangulation across interviews, observations, and stimulated recall data. These procedures enhance the credibility of the findings and support the robustness of the analytical interpretations.

## 7 Conclusion, implications, and limitations

This study set out to examine how psychological factors shape ESL teachers’ integration of AI tools in Ghanaian senior high schools. The findings indicate that AI adoption in instructional contexts is not primarily determined by access to technology or positive perceptions alone, but by teachers’ confidence in their ability to use these tools meaningfully within classroom practice. In particular, teacher self-efficacy emerged as a central mechanism linking perceptions of AI to pedagogical enactment, while contextual constraints influenced how and when this confidence could be translated into instructional action.

By focusing on teachers rather than students, the study contributes to the growing literature on AI in education by highlighting the importance of teacher cognition as a mediating layer between technological potential and instructional reality. The findings suggest that commonly used models such as the Technology Acceptance Model provide only a partial explanation of AI adoption unless complemented by constructs that capture teachers’ sense of competence and their situated engagement with technology. In this sense, the study advances a more integrated understanding of AI use in education as a process shaped by the interaction between perception, confidence, and context.

From a practical perspective, the findings point to the need for a shift in how AI integration is approached in educational settings. Teacher development initiatives should move beyond raising awareness of AI tools toward building practical confidence and pedagogical competence. This includes providing opportunities for teachers to engage with AI in authentic instructional contexts, reflect on their practice, and develop strategies for guiding students’ interaction with AI outputs. In practical terms, this could involve the establishment of peer learning communities in which teachers collaboratively share experiences and experiment with AI-supported activities. Short, hands-on workshops focusing on accessible and low-cost AI tools may further support teachers in developing confidence through guided practice. In addition, in-class coaching or mentoring models, where more experienced or confident teachers demonstrate AI integration strategies in real classroom settings, could help bridge the gap between theoretical understanding and pedagogical enactment, particularly in resource-constrained contexts. Without such support, AI is likely to remain underutilized or confined to surface-level applications.

At the institutional level, the findings highlight the importance of aligning technological initiatives with the realities of classroom practice. Investments in infrastructure, such as reliable internet access, remain essential, but they must be accompanied by sustained professional support that addresses both technical and pedagogical challenges. Policies that assume immediate or widespread adoption of AI without considering teachers’ readiness and contextual constraints risk overestimating the impact of these technologies.

Despite its contributions, the study has several limitations. First, the relatively small sample size limits the transferability of the findings and calls for cautious interpretation beyond similar contexts. Second, while the study draws on multiple qualitative data sources, including interviews, classroom observations, and stimulated recall, the findings remain context-specific and shaped by the conditions under which the data were generated. Third, the focus on a single national context means that the findings may not be directly applicable to other educational settings with different structural and cultural conditions. Future research could address these limitations by conducting comparative studies across contexts and by further examining how different institutional environments shape teachers’ engagement with AI.

The study underscores that the integration of AI in ESL education depends not only on technological innovation but also on the psychological and contextual conditions that shape how these tools are enacted in practice. By foregrounding the role of teacher psychology, the study provides a basis for more grounded and context-sensitive approaches to AI-supported teaching and learning.

## Data Availability

The raw data supporting the conclusions of this article will be made available by the authors, without undue reservation.
