# Supplementary material for: Psychological mechanisms of AI integration in ESL teaching: teacher self-efficacy and classroom practice in Ghanaian senior high schools
Source: Front Psychol. 2026 Jun 10;17:1851325. doi: 10.3389/fpsyg.2026.1851325 (PMC13292424; doi:10.3389/fpsyg.2026.1851325)
Supplement: Supplementary file 1 [file Data_Sheet_1.pdf]

Appendices

Appendix A: Semi-structuredInterview Guide

Section 1: Background Information

1. Can you briefly describe your teaching experience and current role?
2. What subjects and grade levels do you teach?
3. What experience do you have with digital or AI-based tools in teaching?

Section 2: Perceptions of AI (TAM)
4. How do you perceive the usefulness of AI tools in teaching reading and writing?
5. In what ways do you think AI can support student learning?
6. How easy or difficult do you find AI tools to use in your teaching?
7. What challenges have you experienced when using AI tools?

Section 3: Self-Efficacy
8. How confident do you feel using AI tools during your lessons?
9. Can you describe a situation where you felt confident using AI in class?
10. Can you describe a situation where you felt uncertain or hesitant?
11. What factors influence your confidence when using AI tools?

Section 4: Pedagogical Practices
12. How do you typically integrate AI tools into reading or writing lessons?
13. At which stages of instruction (e.g., pre-writing, drafting, feedback) do you use AI?
14. How do students interact with AI tools during your lessons?
15. How do you guide students in using AI outputs?

Section 5: Contextual Factors
16. What institutional or technical factors affect your use of AI?
17. How do time constraints influence your decision to use AI?
18. What kind of support or training would help you use AI more effectively?

Appendix B: Classroom Observation Protocol

1. General Information

- Date:
- School:
- Lesson topic:
- Duration:
- Number of students:

2. AI Use in Instruction

- Type of AI tool used:
- Purpose of use (e.g., feedback, idea generation, modeling):
- Stage of lesson where AI is used:

3. Teacher Practices

- How the teacher introduces the AI tool
- Level of teacher confidence (e.g., fluent, hesitant, interrupted use)
- Integration level:
  - ☐ Instrumental (teacher-centered use)
  - ☐ Partial integration
  - ☐ Full pedagogical integration

4. Student Engagement

- Are students directly interacting with AI? (Yes/No)
- Nature of interaction (e.g., passive viewing, active use, critical discussion)
- Evidence of student engagement

5. Pedagogical Integration

- Alignment with lesson objectives
- Use of AI in instructional sequence (isolated vs embedded)
- Teacher guidance on evaluating AI outputs

6. Contextual Factors

- Internet connectivity issues
- Time constraints
- Classroom management challenges

7. Field Notes
(Open-ended observations of key events, interactions, or notable moments)

Appendix C: Stimulated Recall Interview Guide

Context: Conducted after classroom observation using specific lesson moments.

1. Can you describe what you were trying to achieve at this moment in the lesson?
2. What made you decide to use (or not use) the AI tool here?
3. How confident did you feel during this part of the lesson?
4. What were you thinking while interacting with the AI tool?
5. Were there any concerns or uncertainties at that moment?
6. How did students respond to the activity?
7. Looking back, would you do anything differently? Why?
8. What challenges did you experience during this lesson?
9. How did contextual factors (e.g., time, internet) influence your decisions?
10. How do you evaluate the effectiveness of AI use in this lesson?
